# Single-cell transcriptome profiling of sepsis identifies *HLA-DR*^*low*^*S100A*^*high*^ monocytes with immunosuppressive function

**DOI:** 10.1186/s40779-023-00462-y

**Published:** 2023-06-19

**Authors:** Ren-Qi Yao, Peng-Yue Zhao, Zhi-Xuan Li, Yu-Yang Liu, Li-Yu Zheng, Yu Duan, Lu Wang, Rong-Li Yang, Hong-Jun Kang, Ji-Wei Hao, Jing-Yan Li, Ning Dong, Yao Wu, Xiao-Hui Du, Feng Zhu, Chao Ren, Guo-Sheng Wu, Zhao-Fan Xia, Yong-Ming Yao

**Affiliations:** 1 Translational Medicine Research Center, Medical Innovation Research Division and the Fourth Medical Center of Chinese PLA General Hospital, Beijing, 100853 China; 2 Department of Burn Surgery, the First Affiliated Hospital of Naval Medical University, Shanghai, 200433 China; 3 Research Unit of Key Techniques for Treatment of Burns and Combined Burns and Trauma Injury, Chinese Academy of Medical Sciences, Beijing, 100730 China; 4 Department of General Surgery, the First Medical Center of Chinese PLA General Hospital, Beijing, 100853 China; 5 Department of Neurosurgery, the First Medical Center of Chinese PLA General Hospital, Beijing, 100853 China; 6 Department of Critical Care Medicine, the First Medical Center of Chinese PLA General Hospital, Beijing, 100853 China; 7 Intensive Care Unit, Dalian Municipal Central Hospital Affiliated Dalian University of Technology, Dalian, 116033 Liaoning China; 8 Department of Emergency, the Second Hospital of Hebei Medical University, Shijiazhuang, 050000 China; 9 Department of Pulmonary and Critical Care Medicine, Beijing Chaoyang Hospital, Capital Medical University, Beijing, 100020 China

**Keywords:** Single-cell analysis, Sepsis, Immunosuppression, S100A, Human leukocyte antigen DR (HLA-DR), Monocytes, Myeloid-derived suppressor cells (MDSCs), Paquinimod

## Abstract

**Background:**

Sustained yet intractable immunosuppression is commonly observed in septic patients, resulting in aggravated clinical outcomes. However, due to the substantial heterogeneity within septic patients, precise indicators in deciphering clinical trajectories and immunological alterations for septic patients remain largely lacking.

**Methods:**

We adopted cross-species, single-cell RNA sequencing (scRNA-seq) analysis based on two published datasets containing circulating immune cell profile of septic patients as well as immune cell atlas of murine model of sepsis. Flow cytometry, laser scanning confocal microscopy (LSCM) imaging and Western blotting were applied to identify the presence of S100A9^+^ monocytes at protein level. To interrogate the immunosuppressive function of this subset, splenic monocytes isolated from septic wild-type or *S100a9*^*−/−*^ mice were co-cultured with naïve CD4^+^ T cells, followed by proliferative assay. Pharmacological inhibition of S100A9 was implemented using Paquinimod via oral gavage.

**Results:**

ScRNA-seq analysis of human sepsis revealed substantial heterogeneity in monocyte compartments following the onset of sepsis, for which distinct monocyte subsets were enriched in disparate subclusters of septic patients. We identified a unique monocyte subset characterized by high expression of S100A family genes and low expression of human leukocyte antigen DR (HLA-DR), which were prominently enriched in septic patients and might exert immunosuppressive function. By combining single-cell transcriptomics of murine model of sepsis with in vivo experiments, we uncovered a similar subtype of monocyte significantly associated with late sepsis and immunocompromised status of septic mice, corresponding to *HLA-DR*^*low*^*S100A*^*high*^ monocytes in human sepsis. Moreover, we found that S100A9^+^ monocytes exhibited profound immunosuppressive function on CD4^+^ T cell immune response and blockade of S100A9 using Paquinimod could partially reverse sepsis-induced immunosuppression.

**Conclusions:**

This study identifies *HLA-DR*^*low*^*S100A*^*high*^ monocytes correlated with immunosuppressive state upon septic challenge, inhibition of which can markedly mitigate sepsis-induced immune depression, thereby providing a novel therapeutic strategy for the management of sepsis.

**Supplementary Information:**

The online version contains supplementary material available at 10.1186/s40779-023-00462-y.

## Background

Sepsis refers to a life-threatening organ dysfunction due to a dysregulated host response to infection based on the current definitions [1]. The recent Global Burden of Diseases study estimated 50 million cases of sepsis worldwide in 2017, with roughly 11 million sepsis-related mortality annually, rendering sepsis as one of the leading causes of critical illness and death [2]. Since the incidence of sepsis increases rapidly, leading to immense societal and economic costs, the World Health Organization highlights sepsis as a global healthcare priority [3, 4]. Nevertheless, decades of attempts regarding effective treatment for sepsis don’t yield encouraging results, as evidenced by numerous but unsuccessful randomized controlled trials (RCTs) [5]. To date, remedies for septic patients appear to be limited, including source control, fluid resuscitation, and supportive measurements on organ dysfunction [6]. This divergency is largely attributed to the substantial heterogeneity among septic patients, prompting researchers to consider the possibility for subgrouping patients with sepsis on the basis of clinical and laboratory features (phenotypes) as well as targetable pathobiological markers (endotypes) [7, 8]. To achieve this, an in-depth understanding of pathogenesis and pathophysiological alterations of sepsis seems to be the prerequisite.

Sepsis elicits a complex response simultaneously exhibiting proinflammatory and anti-inflammatory features, but presenting with an impaired homeostasis [9]. An initial hyperinflammatory response is closely associated with tissue damage and organ dysfunction, whereas concurrent or delayed anti-inflammatory response greatly facilitate sustained immunosuppression, thereby contributing to susceptibility to nosocomial infections, increased rehospitalization, and even elevated late mortality [4, 10]. Sepsis-induced immunodeficiency is commonly and consistently observed in septic patients, it remains less evident and unrecognizable in many cases since there is lack of standardization in evaluating immune functional status for patients who manifest diverse yet context-specific immunological alterations [11]. Lymphocyte counts and expression of monocyte human leukocyte antigen DR (mHLA-DR) have been widely applied for the assessment of immune function among septic patients, but they have certain limitations. Although lymphocyte count is easily obtained, its specificity is relatively low due to many influencing factors [12, 13]. Concurrently, the early warning thresholds of mHLA-DR remain inconclusive, which are varying across disparate cohorts [14]. Therefore, absence of efficient immune monitoring approaches and indicators obviously hinders the development of tailored immunotherapies, in association with the consecutive failure of RCTs and invalid subgrouping strategies [11]. This dilemma further highlights the urgent need for precise analyses using high-resolution techniques that are capable of deciphering clinical trajectories and immunological changes in septic patients.

Single-cell RNA sequencing (scRNA-seq) represents a robust approach to exploring previously undefined cell types and states [15]. Nowadays, scRNA-seq has been increasingly adopted in resolving heterogeneity within immune cell subset and identifying disease-specific cell signatures in multiple human diseases, including various malignancies, autoimmune disorders, inflammatory diseases, and coronavirus disease 2019 (COVID-19) [16–19]. As sepsis-induced immunosuppression is mainly due to dysfunction of various immune cells intrinsically, scRNA-seq seems to represent an optimal candidate. Correspondingly, several studies focused on immune cell profile of patients with sepsis at single-cell level [20–22]. Of note, a pioneering single-cell study by Reyes et al. [20] incorporated three independent cohorts of sepsis containing five clinical categories, in which they identified a unique subtype of circulating CD14^+^ monocytes that presented with immunosuppressive function. Meanwhile, our previous study applied scRNA-seq analysis on murine model of sepsis using CD45^+^ cells derived from multiple tissue compartments of cecal ligation and puncture (CLP)-induced septic mice [23]. In this study, we found a subset of conventional dendritic cell exhibiting high expression of immunoregulatory molecules, namely ‘mregDC’, which was markedly upregulated in the hyperinflammatory stage of sepsis. Given that, these two datasets might serve as ideal resources in guiding future research in terms of sepsis immunology.

By carrying out a cross-species, secondary analysis using both human and murine single-cell datasets in sepsis, we investigated immune cell composition associated with sepsis immunosuppression and progression. Meanwhile, we characterized the unique features and functions of the immunosuppressive monocyte subtypes with the help of various ex vivo and in vivo validating experiments. These findings enriched our understanding on the cellular and molecular basis of sepsis-induced immunosuppression, providing a novel yet potent indicator for the monitoring and treatment of immune dysregulation in the setting of sepsis.

## Methods

### Mice

C57BL/6J mice, 6–8 weeks, were purchased from the Laboratory Animal Science of Chinese Academy of Medical Sciences, Beijing, China. *S100*a*9*^*−/−*^ mice was purchased from Cyagen Transgenic Animal Center, Guangzhou, China. Merely male mice were adopted for the subsequent experiments, which were housed in specific pathogen-free conditions. All experimental procedures were approved by the Scientific Investigation Board of Chinese PLA General Hospital (SYXK2020-0015), Beijing, China.

### Murine model of sepsis

Mouse model of sepsis was reproduced by CLP surgery. Anesthesia of mice was carried out using 5% chloral hydrate, followed by disinfection of skin. Thereafter, abdominal incision was undergone to sufficiently expose the cecum prior to ligation below the ileocecal valve. Then, cecum was punctured using 16-gauge needle and fractional feces was extradited through compressing the cecum. After close of incision, CLP mice were subcutaneously injected with 1 ml 0.9% normal saline for fluid resuscitation. Mice in the sham group were merely performed cecum exposure without undergoing ligation and puncture procedures. For in vivo experiments, mice undergoing CLP surgery were sacrificed through euthanizing with CO_2_ after disparate time points (0, 24, and 72 h), followed by subsequent assays. To investigate the effect of Paquinimod, mice were randomly yet equally divided into three groups: sham group, CLP group, CLP + Paquinimod group. CLP mice were given Paquinimod [(10 mg/(kg·d)] by oral gavage for 3 d.

### Isolation of peripheral blood mononuclear cells (PBMCs) and monocytes

PBMCs were isolated by use of an isolation kit complying with the manufacturer’s protocols. Briefly, whole blood of mice was collected from retro-orbital bleeding and diluted using phosphate buffer solution (PBS) supplemented with ethylene diamine tetraacetic acid. Thereafter, suspensions were gently added to the surface of lymphoprep, followed by centrifugation for 20 min at 500 g. Next, interphase PBMCs were obtained, washed and resuspended using PBS for subsequent assay. Circulating monocytes were isolated using a mouse monocyte isolation kit. Whole blood was lysed on ice for 15 min, and resuspended in PBS containing 2% fetal bovine serum. Selection cocktail was pre-mixed for 5 min, followed by adding to the cell suspension and incubating for 5 min at 4 °C. Magnetic beads were added to the sample and incubated for 5 min at 4 °C. Then, tube containing cell suspension was topped up and placed into the magnet for incubation for 3 min. Enriched monocyte suspension was pipetted into a new tube and washed twice prior to subsequent assay.

### scRNA-seq analysis

Publicly available scRNA-seq datasets were downloaded from Gene Expression Omnibus (GSE151263) and Single Cell Portal (SCP548) using accession code and obtained from our previous study. scRNA-seq analyses were performed by use of R software and the ‘Seurat’ package. During the quality control, cells with detected genes < 200 and mitochondrial content > 10% were removed from the subsequent analysis. Doublets or multiplets were filtered out based on gene expression signatures. Filtered unique molecular identifiers were then normalized by adopting the ‘NormalizeData’ function (normalization method = ‘logNormalize’, scaling factor = 10,000).

### Unsupervised clustering analysis

To identify the highly variable genes, linear dimensionality reduction was employed using ‘FindVariableGenes’ function with default parameters. Principal component analysis was performed on the basis of the highly variable genes by applying ‘RunPCA’ function, and ElbowPlot was used to measure the optimal numbers of principal components. Bidimensional coordinates of single cell were obtained with the help of ‘RunUMAP’ function (perplexity value = 30). To visualize sample in uniform manifold approximation and projection (UMAP) plot, we thereby carried out cell clustering analysis using ‘FindClusters’ based on the same PCs in the ‘RunUMAP’ function.

### Identification of differentially expressed genes (DEGs)

DEGs across subclusters were identified by ‘FindAllMarkers’ function based on the normalized data. *P-*values were adjusted using Bonferroni correction. Correspondingly, differential expression on each subpopulation was performed by use of Wilcoxon rank sum test in ‘Seurat’, which was shown by heatmap. Upregulated DEGs of monocyte subtypes with *P*-value less than 0.05 as well as log_2_ fold change exceeding 0.5 were compared and visualized by volcano plots.

### Pseudotime trajectory analysis

Pseudo-time trajectory analyses were employed using ‘Monocle2’ based on signature genes identified by ‘DifferentialGeneTest’ function. Generalized additive models were constructed to generate the average expression of isoforms. The formula of monocle’s generalized additive model was used: *E*(*Y*) = *s*[*Ψt*(*bx*, *si*)] + ϵ, where *Ψt*(*bx*, *si*) and ϵ represented the pseudo-time and normally distributed error term, respectively. Function *s* was a cubic smoothing function. The developmental trajectories among monocyte subsets were carried out using ‘Monocle’ with default parameters.

### Cell–cell interaction network analysis

By adopting ‘CellPhoneDB’, cell–cell interacting analysis was conducted to predict functional ligand-receptor pairs. The receptor-ligand interactions were analyzed corresponding to receptors expressing in one immune cell type and an isogenic ligand in another cell type. The interaction intensity across cell subclusters was measured using permutation test. Ligand-receptor partners with interacting intensity more than 10 with *P-*value less than 0.01 were considered as significant pairs in mediating cell–cell communications.

### Flow cytometry

To measure expressions of surface molecules, cells were stained with FITC anti-mouse major histocompatibility complex (MHC)-II antibody (1:200; Biolegend, 107606), BV510 anti-mouse CD3 antibody (1:200; Biolegend, 100234), FITC anti-mouse CD4 antibody (1:200; Biolegend, 100405) or APC anti-mouse CD25 antibody (1:200; Biolegend, 101909) in FACS buffer for 40 min. For intracellular staining, cells were permeabilized, followed by fixation using the Foxp3 staining buffer kit prior to staining with PE anti-mouse Foxp3 antibody (1:100; Biolegend, 320007), BV421 anti-mouse Foxp3 antibody (1:100; Biolegend, 126419), or PE-conjugated S100A9 antibody (1:100; Cell Signaling Technology, 93941). Flow cytometry analyses were performed on a LSR II instrument and raw data were retrieved. Results were analyzed by use of FlowJo software version 10.0.

### Histological examination

The dissected samples were fixed with 4% paraformaldehyde, followed by embedding in paraffin blocks. Cryosections were then deparaffinized and stained with hematoxylin–eosin prior to observation through microscopy. Two histologists separately examined the histological changes of sections, with unawareness of the grouping.

### Measurement of cytokine levels

To determine contents of interleukin (IL)-2, IL-4, IL-10, interferon (IFN)-γ, and transforming growth factor (TGF)-β, supernatants or plasma were diluted and assayed by enzyme-linked immunosorbent assay (ELISA) kits. The results were assayed and analyzed using ELISA plate reader.

### Western blotting

After mixing with 5 × SDS-loading buffer and denaturation at 95 °C, prepared samples were loaded onto and separated by 8–12% SDS polyacrylamide gel electrophoresis. Thereafter, the gels were transferred onto a polyvinylidene fluoride membranes, followed by blocking using 10% evaporated milk. Membranes were incubated with rabbit anti-S100A9 antibody (1:1000; Abcam, ab242945) and anti-rabbit HRP-conjugated secondary antibody. β-actin was served as a loading control. The blots were visualized under electrochemiluminescence system.

### Laser scanning confocal microscopy (LSCM)

Cell samples were fixed with the use of 4% paraformaldehyde and permeabilizated using 0.3% Triton X-100 for 15 min. Cells were washed and blocked with 1% bovine serum albumin for 1 h. Afterwards, cells were stained with anti-S100A9 antibody (1:200; Cell Signaling Technology, 73425) followed by incubation with fluorochrome-conjugated secondary antibody after PBS wash for 3 times. Finally, cells were stained with 4′, 6′-diamidino-2-phenylindole prior to mounting onto slides. The slides were observed under LSCM.

### CD4^+^ T cell assay

The splenic CD4^+^ T lymphocytes were isolated from the mononuclear cells using magnetic cell sorting system (Miltenyi Biotech, Bergisch Gladbach, Germany) according to the manufacturer’s instructions. Mononuclear cells were incubated with CD4^+^ microbeads (20 μl per 10^7^ cells) for 15 min at 4 °C, followed by collection of splenic CD4^+^ T lymphocytes via magnetic separation. CD4^+^ T lymphocytes were resuspended with complete RPMI 1640 medium (10% fetal bovine serum, 100 U/ml penicillin, and 100 μg/ml streptomycin), and then inoculated in 96-well plates at 4 × 10^5^ cells per well to be cultured with the stimulation of both soluble CD3 (1 μg/ml, BioLegend, 100339, San Diego, CA) and CD28 (1 μg/ml, BioLegend, 102115, San Diego, CA) for 24 h in 5% CO_2_, 37 °C incubator. Monocytes were then co-cultured with T cells at 1:1 ratio for another 72 h. To assess proliferative activity of T cells, we stained CD4^+^ T cells with carboxyl fluorescein succinyl ester staining in line with the manufacturer’s instructions. Upon harvesting, supernatants and CD4^+^ T cells were collected and analyzed by use of flow cytometry. For ex vivo experiments, monocytes from spleen of wild-type (WT) or *S100a9*^*−/−*^ mice undergoing sham or CLP surgery for 72 h were isolated, followed by co-culture with activated naïve CD4^+^ T cells isolated from unmanipulated murine spleens.

### Statistical analysis

Statistical analyses were conducted using SPSS software version 23.0 and GraphPad Prism 8. Measurement data were expressed as mean ± standard deviation (SD). Statistics were calculated using the unpaired student’s *t* test for comparisons between groups. One-way or two-way analysis of variance (ANOVA) was also carried out for multiple comparisons, accompanied by the Tukey HSD test for post-hoc comparisons. Numeration data were expressed as absolute values with percentages, for which Pearson’s correlation test was used for statistical analysis. The Kaplan–Meier analysis was applied with regard to survival experiments, and log-rank test was used for testifying the differences between survival curves. Two tailed *P*-value less than 0.05 were considered to be statistically significant.

## Results

### Single-cell atlas of PBMCs from septic patients

**Fig. 1:**
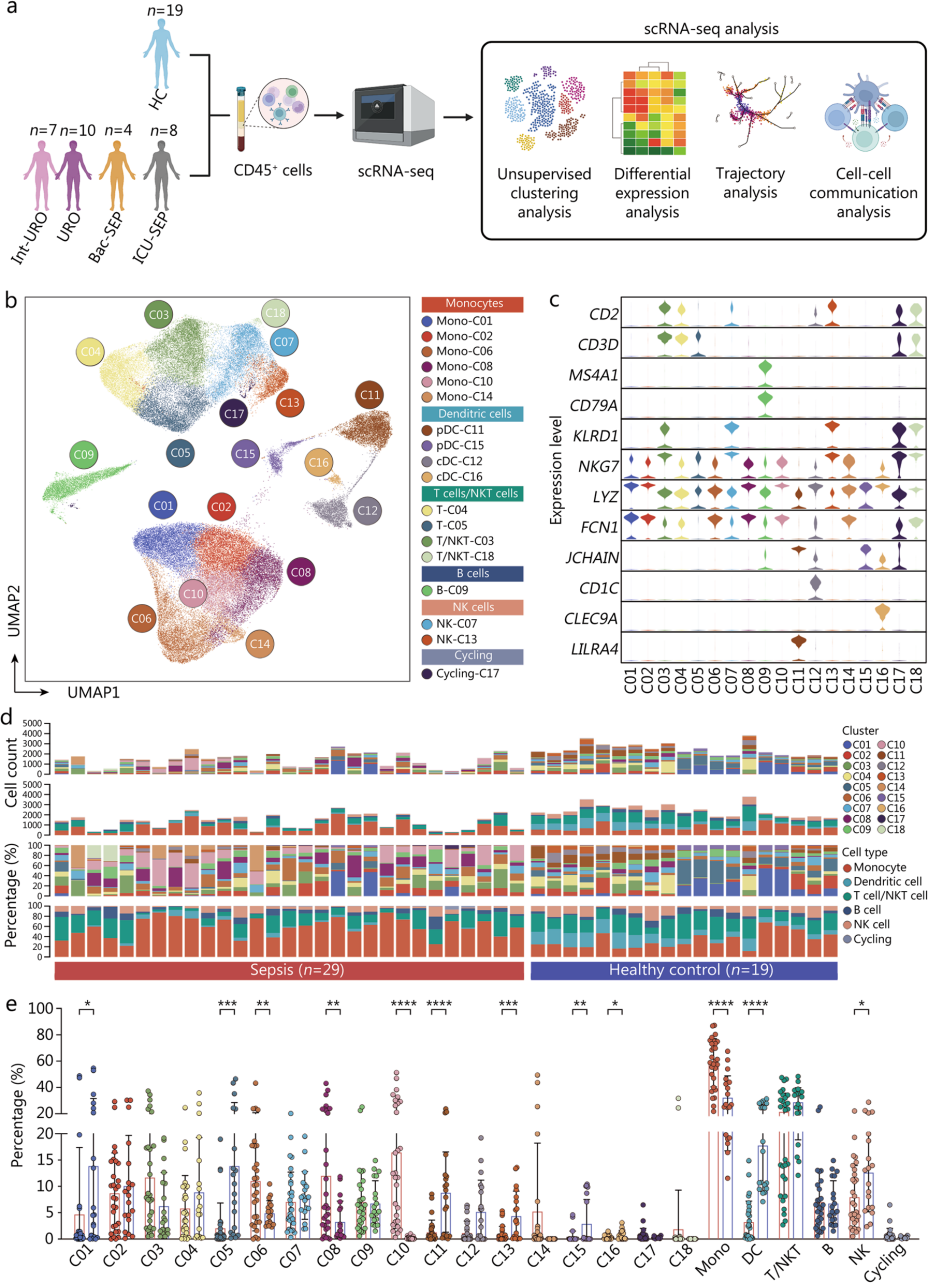
Single-cell atlas of PBMCs from septic patients. **a** Schematic workflow of the current study. **b** UMAP visualization of scRNA-seq profiles of 48 samples (29 septic patients and 19 HC) displayed the annotation and color codes for 18 immune cell subclusters. **c** Violin plots indicated expression level of canonical annotation marker gene. **d** Proportion and absolute counts of each subcluster and each immune cell type across enrolled participants. **e** Quantitative bar charts showed the comparison of percentage of each subcluster and each immune cell type between patients with sepsis and HC. Statistics were analyzed by unpaired two-sided Student’s *t* test. Data are shown as means ± SD. ^*^P < 0.05, ^**^P < 0.01, ^***^P < 0.001, ^****^P < 0.0001. PBMCs peripheral blood mononuclear cells, UMAP uniform manifold approximation and projection, scRNA-seq single-cell RNA sequencing, HC healthy controls, SD standard deviation, Mono monocyte, DC dendritic cell, NK natural killer

To delineate immune response between septic patients and healthy individuals, we collected an online available dataset containing scRNA-seq data of PBMCs from three clinical cohorts with a total of 65 participants [20]. Specifically, other than 19 individuals designated as healthy controls, these cohorts included patients with disparate clinical entities, including urinary tract infection (UTI) with leukocytosis (Leuk-UTI), UTI with intermediate organ dysfunction (Int-URO), UTI with evident organ dysfunction (URO), bacterial sepsis in hospital wards (Bac-SEP), and admission to the intensive care unit (ICU) with sepsis (ICU-SEP) or without sepsis (ICU-NoSEP). We combined patients with Int-URO, URO, Bac-SEP, and ICU-SEP into patients with sepsis (*n* = 29) according to Sepsis 3.0 definition (sequential organ failure assessment score ≥ 2 along with a confirmed or suspected infection), which were subjected to subsequent analysis with healthy controls (*n* = 19). Clinical characteristics of all enrolled patients were summarized in Additional file 1: Table S1. Schematic workflow of this study is summarized in Fig. 1a.

After removing doublets and filtering out cells of low quality, 84,144 cells were eligible for the subsequent analysis, with 22,858 detected genes. By adopting UMAP analysis, 18 cell clusters were identified consequently and revealed highly disease-specific clusters after separate visualization of cells from either septic cases or healthy individuals (Fig. 1b, Additional file 1: Fig. S1a). Cells were classified into 6 immune cell subtypes which were further annotated on the basis of the expression of marker genes: monocytes (*LYZ* and *FCN1*), dendritic cells (DCs) (*JCHAIN* and *LILRA4* for plasmacytoid DCs, *CLEC9A* and *CD1C* for type 1 conventional DC and type 2 conventional DC, respectively), T cells (*CD2* and *CD3D*), B cells (*MS4A1* and *CD79A*), natural killer (NK) cells (*KLRD1* and *NKG7*), and cycling cells (Fig. 1c, Additional file 1: Fig. S1b). As shown in Fig. 1d, substantial enrichment of monocytes could be consistently noticed in septic patients, especially for C10. Meanwhile, we found that the majority of monocyte subclusters’ proportion was significantly elevated in septic patients compared with that of healthy controls, including C06, C08, and C10, and so did the overall monocyte percentage (Fig. 1e). Of note, C01 showed marked reduction in septic patients, manifesting as divergent tendency with other subsets. Additionally, substantial decreases could be observed in proportions of DCs and NK cells among patients with sepsis. Moreover, we probed the correlations across disparate immune cell subsets (Additional file 1: Fig. S1c). Intriguingly, C10, a monocyte subset expressing upregulated level of S100A8, was significantly correlated with the reduction of multiple immune cell subclusters, including C01 (monocytes), C13 (NK cells), C04 and C05 (T cells), C11 and C15 (plasmacytoid DCs), implying its potential immunoregulatory function. Taken together, these findings suggest a dramatic alteration in peripheral immune response upon septic insults, especially for monocyte subtypes.

### Enrichment of distinct monocyte subtypes in septic patients

**Fig. 2:**
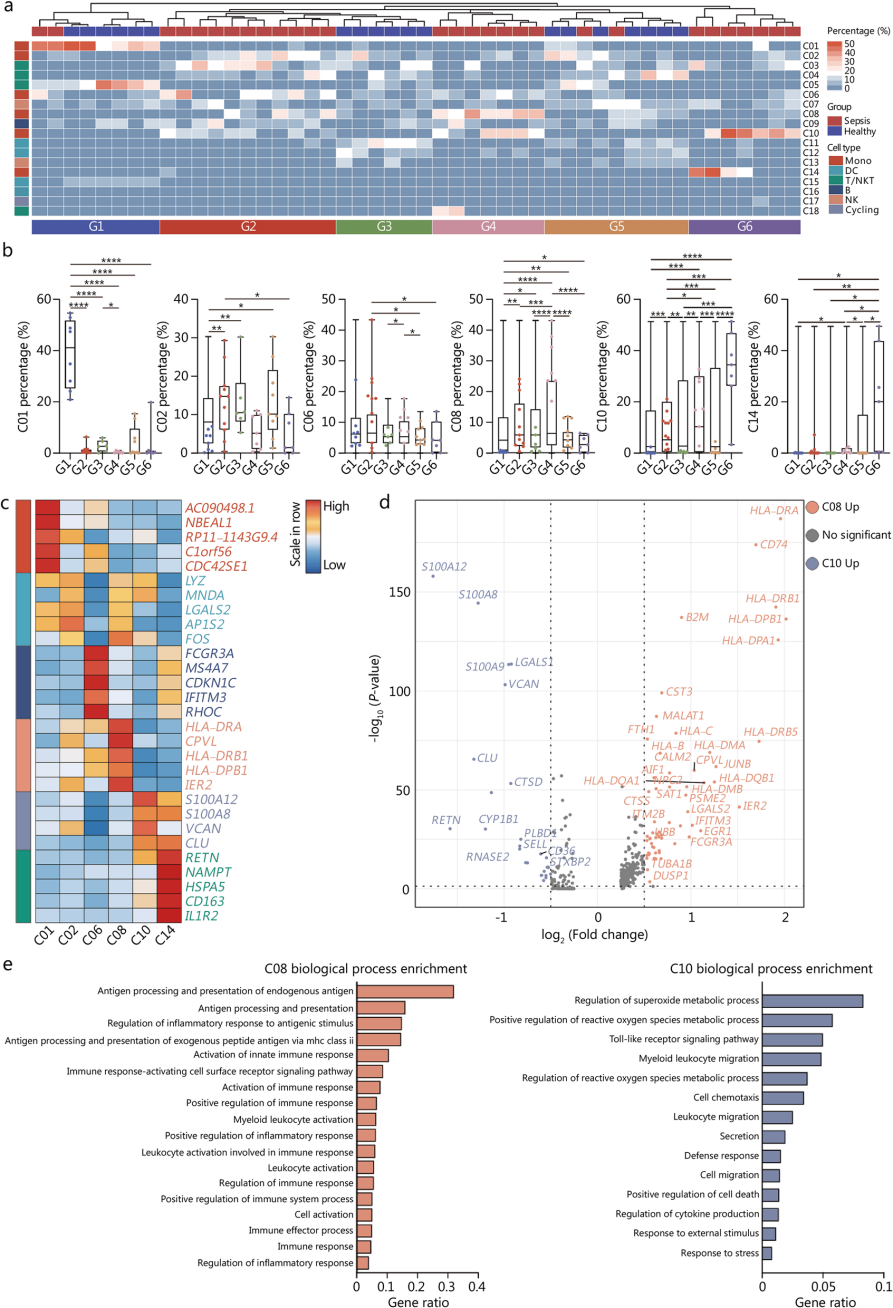
Enrichment of distinct monocyte subtypes in septic patients. **a** Histogram of unsupervised clustering analysis was divided enrolled participants into 6 clusters based on enrichment of disparate immune cell groups. **b** Quantitative bar charts were compared proportion of monocyte subsets across different clusters of individuals. **c** Heatmap showed relative expression level of top 5 DEGs among subclustered monocyte subsets. **d** Volcano plot displayed upregulation of DEGs regarding C08 vs. C10. **e** Bar graph listed the enriched biological processes in C08 (left panel) and C10 (right panel) by GO analysis. One-way ANOVA with Tukey HSD test was applied to calculate statistics. Data are shown as means ± SD. ^*^P < 0.05, ^**^P < 0.01, ^***^P < 0.001, ^****^P < 0.0001. DEGs differentially expressed genes, Mono monocyte, DC dendritic cell, NK natural killer, ANOVA analysis of variance, SD standard deviation

Since severity of illness reflected by sequential organ failure assessment or acute physiology and chronic health evaluation II remains largely insufficient for evaluating the disease progression, deciphering common immune cell features across septic patients may shed light on the identification of distinct phenotypes based on immune signatures, thereby facilitating the establishment of tailored immunomodulatory regime [8, 9]. In this regard, we conducted unsupervised clustering analysis in accordance with enrichment of disparate immune cell subsets in each participant, which yield 6 clusters (G1 to G6), with distinct immune cell features. As shown in Fig. 2a, healthy individuals were dominant in G1, G3, and G5 subsets, with only a fraction of patients with sepsis, whereas all enrolled cohorts from G2, G4, and G6 solely contained septic patients. For cluster of G2, proportions of two monocyte subsets (C02 and C06) were predominantly enriched. Strikingly, C08 and C10 were primarily infiltered in G4 cluster, while substantial enrichment of C10 and C14 could be observed in G6 (Fig. 2b). Detailed composition of clinical entities for each subset was summarized in Additional file 1: Table S2, in which disease categories of septic patients assigned in G2, G4, and G6 remained relatively varied. These data indicate that enrichment of monocyte subtypes is largely disparate across septic patients, implicating the potential of monocyte subsets in discriminating endotypes of septic cases.

Based on marker gene expression, we performed annotation on 6 monocyte subtypes in order to further clarify their contributions to disease progression (Fig. 2c, Additional file 1: Fig. S2a). Monocytes from C01 and C02 expressed *CD14*, with low or no expression of *FCGR3A*, corresponding to classical monocytes. C06 expressed transcripts indicative of non-classical monocytes, as evidenced by high level of *FCGR3A* and diminished expression of *CD14*. Monocytes from C08 were characterized by upregulated level of *HLA-DRA* and *HLA-DRB1*, which closely resembled *CD14*^*+*^*HLA-DR*^*high*^ inflammatory monocyte. C10 monocytes exhibited high expression of S100A family genes, including *S100A8* and *S100A12*, along with low expression of *HLA-DR*, reminiscent of *HLA-DR*^*low*^*S100A*^*high*^ monocytes in severe COVID-19 [24]. Notably, this subset showed obvious activation of genes that were related to worsening clinical outcomes in septic patients, including *PLAC8*, *RETN* and *CLU* [25]. Meanwhile, cells form cluster of C14 closely resembled *HLA-DR*^*low*^*CD163*^*high*^ monocytes, since it displayed enhanced expression of *CD163* with reduction in *HLA-DR* expression, in association with the phenotype of anti-inflammatory macrophage [26, 27]. By comparing upregulated DEGs regarding C08 versus C10, it further substantiated the dysfunctional phenotype of *HLA-DR*^*low*^*S100A*^*high*^ monocytes, as shown by dampened expression of functional molecules and significant upregulation of damage-associated molecular patterns, including S100A family genes and *LGALS1*, which greatly prompted programmed cell death and immune disorder (Fig. 2d). As depicted in Fig. 2e, the GO functional annotation of upregulated genes from *HLA-DR*^*low*^*S100A*^*high*^ monocytes further underpinned this point, for biological processes including defense response, positive regulation of reactive oxygen species metabolic process, Toll-like receptor (TLR) signaling pathway, and positive regulation of cell death were predominantly enriched in this subset, reminiscent of myeloid-derived suppressor cells (MDSCs) signatures [28]. To investigate and characterize specific ligand-receptor interactions across disparate immune cell subclusters, we performed cell–cell communication analysis using ‘CellPhoneDB’. Interestingly, immune inhibition and immune checkpoint-related interactions between *HLA-DR*^*low*^*S100A*^*high*^ monocytes and other immune cells were identified and visualized, including TGFB1-TGFBR3, LGALS9-HAVCR2, TNFRSF13B-TNFSF13B, and HLA-LILRB, hinting a potential immunosuppressive capacity of *HLA-DR*^*low*^*S100A*^*high*^ monocytes (Additional file 1: Fig. S2b). We further probed the phenotypic alterations as well as temporal dynamics within monocyte subsets using pseudo-time and trajectory analyses. As expected, the putative trajectories based on cell states showed that both *HLA-DR*^*low*^*S100A*^*high*^ and *HLA-DR*^*low*^*CD163*^*high*^ monocytes dominated in the relatively late phase along pseudo-time (Additional file 1: Fig. S2c, d), whereas classical monocytes presented at the initial state. To further validate the conserved presence of *HLA-DR*^*low*^*S100A*^*high*^ monocytes in human sepsis, we interrogated monocyte heterogeneity using another dataset containing PBMCs from septic patients with or without acute respiratory distress syndrome (Additional file 1: Fig. S3a) [21]. Consequently, clustering analysis of circulating monocytes yielded 7 subsets, in which C01 and C02 expressed suggestive of *HLA-DR*^*low*^*S100A*^*high*^ monocytes, including *S100A8*, *S100A9*, and *S100A12* (Additional file 1: Fig. S3b, c). Monocytes from C01 and C02 also expressed high levels of *VCAN*, *RETN*, and *CTSD*, all of which presented with the top ranking DEGs in *HLA-DR*^*low*^*S100A*^*high*^ monocytes. Collectively, we identify a unique yet conserved monocyte subset that is prominently enriched in septic patients, and it might play an essential role in predicting immunocompromised state and sepsis progression.

### Predominance of *S100a*^*high*^ monocytes in late sepsis

**Fig. 3:**
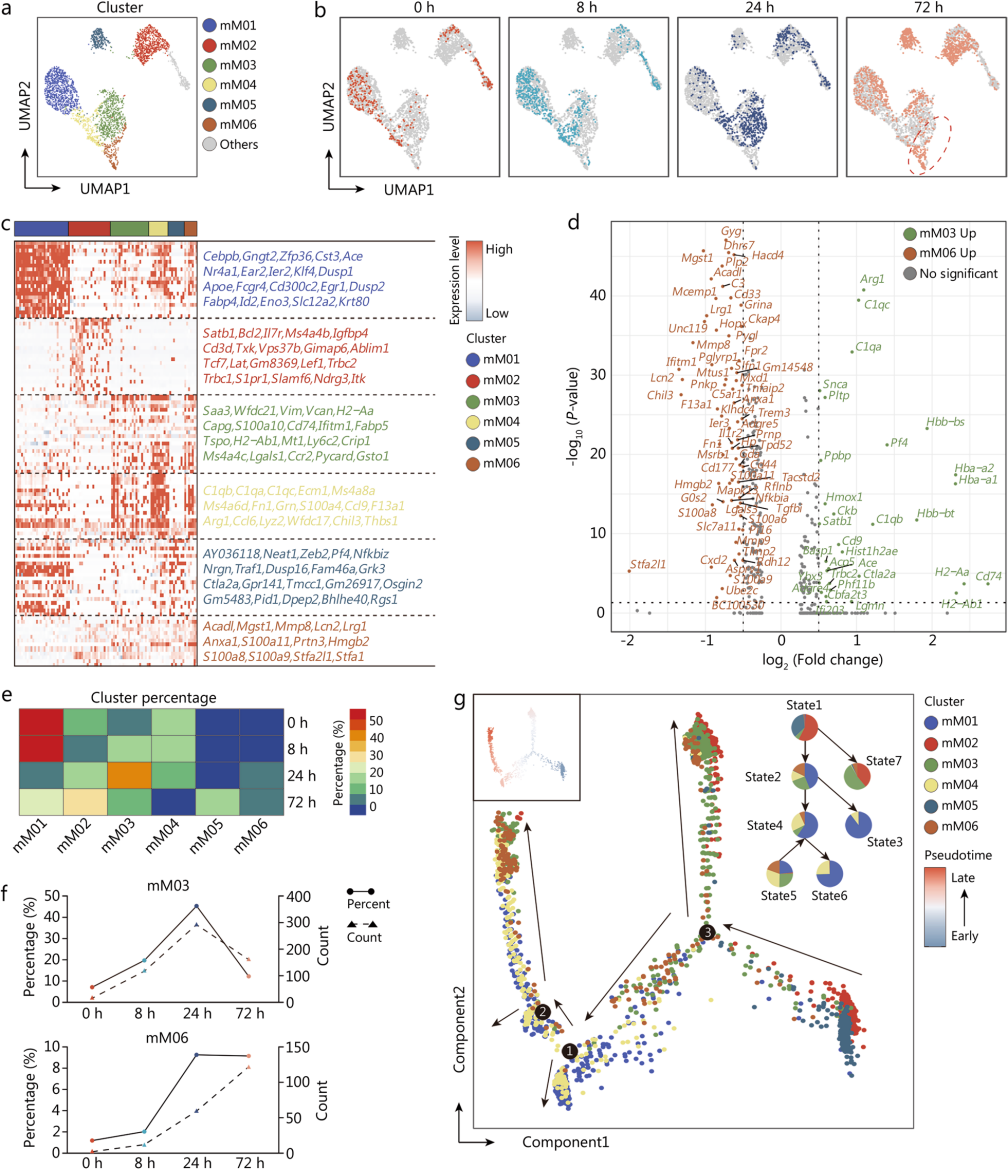
Predominance of *S100a*^*high*^ monocytes in late sepsis. **a** UMAP plot showed subclusters of circulating monocytes in mouse model of sepsis. **b** UMAP plots of monocyte subsets, across disparate sampling time points after CLP operation. **c** Histogram showed the relative expression of DEGs among all monocyte subpopulations. **d** Volcano plot compared upregulation of DEGs regarding mM03 vs. mM06. **e** Heatmaps displayed relative enrichment of each monocyte subcluster during the course of sepsis. **f** Curve plots showed proportion of mM03 (upper panel) and mM06 (lower panel) at distinct timepoints after CLP surgery. **g** The developmental trajectory of monocytes was colored-coded by the clusters and pseudo-time. Putative trajectory for cell transition states of monocyte, with proportion of each subcluster (upper right panel). UMAP uniform manifold approximation and projection, CLP cecal ligation and puncture, DEGs differentially expressed genes

To explore the correlation of *HLA-DR*^*low*^*S100A*^*high*^ with disease progression as well as sepsis-induced immunosuppression, we conducted a parallel, cross-species study on experimental model of sepsis using scRNA-seq data from our recently published resource, in which circulating CD45^+^ cells from mice undergoing sham surgery (0 h) or CLP operation at various intervals (8, 24, and 72 h) were isolated and subjected to scRNA-seq [23]. Corresponding to expression of canonical markers, including *Csf1r* and *Ly86*, peripheral blood monocytes were visualized using UMAP dimensionality reduction, yielding a total of 7 subclusters (Fig. 3a). Meanwhile, UMAP clusters showing quantitative alterations of monocytes during the course of sepsis were independently visualized (Fig. 3b). Thereafter, we performed annotation on each monocyte subset on the basis of DEGs across subclusters (Fig. 3c). Comparison of upregulated genes of mM03 vs. mM06 further substantiated that mM03 and mM06 were corresponded to *CD14*^*+*^*HLA-DR*^*high*^ and *HLA-DR*^*low*^*S100A*^*high*^ monocytes in human sepsis, respectively (Fig. 3d). We therefore plotted the curve reflecting the percentage and absolute counts of mM03 and mM06 at various sampling intervals after septic induction. As expected, mM03 was noted to dominate at early phase of sepsis, whereas substantial enrichment of mM06 was found at 72 h post-operation (Fig. 3e, f). Likewise, pseudo-time and trajectory analysis revealed that *S100a*^*high*^ monocytes presented at late stage along pseudo-time, in consistent with the observation in human sepsis (Fig. 3g). In support of our results in peripheral blood, scRNA-seq analysis on splenic CD45^+^ cells derived from septic mice simultaneously identified the appearance of *S100a*^*high*^ monocytes, implicating its possible impact on organ-specific immune response to septic insults (Additional file 1: Fig. S4a, b).

**Fig. 4:**
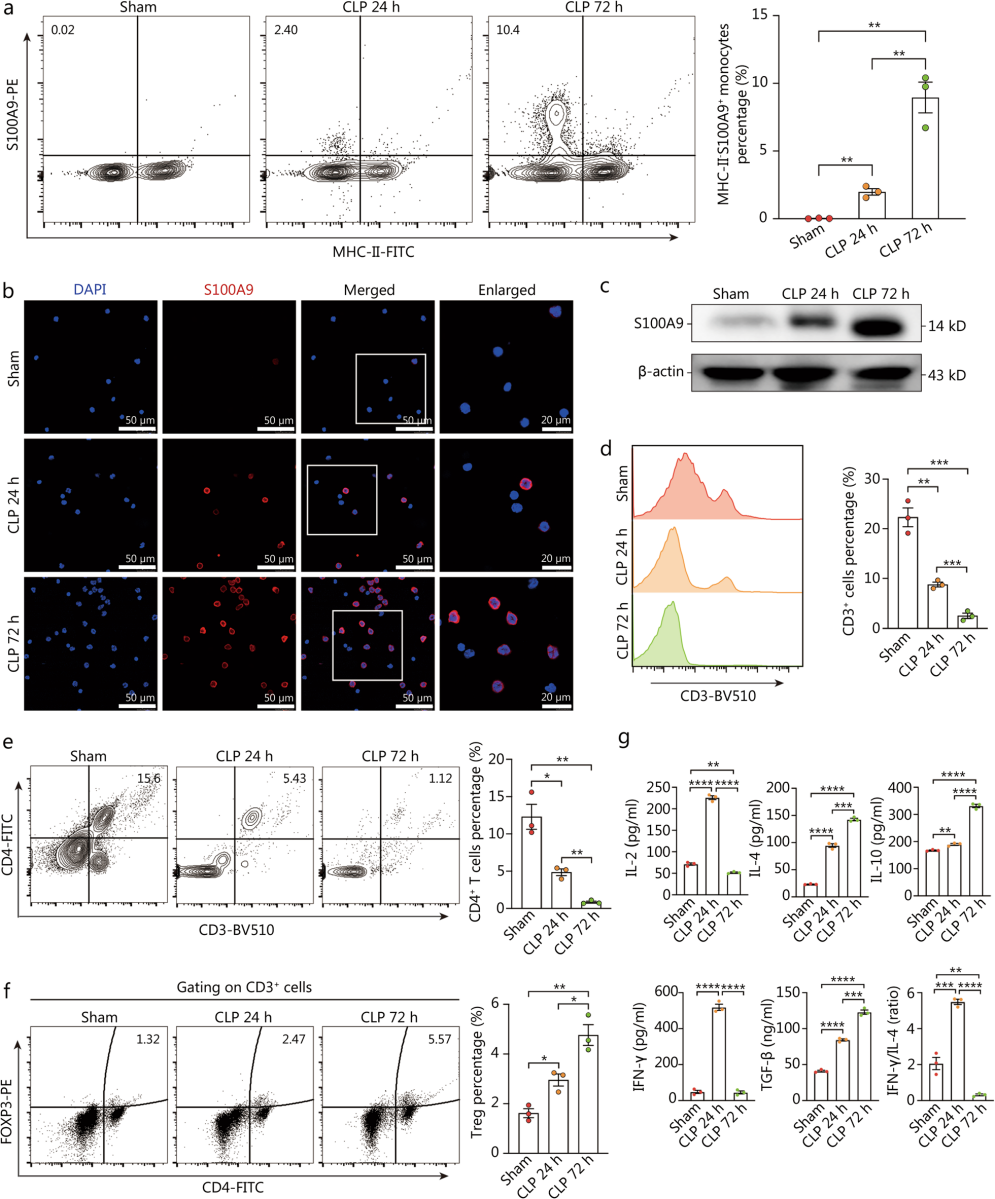
MHC-II^−^S100A9^+^ monocytes are associated with immunocompromised state in late sepsis. **a–c** At different intervals after CLP surgery (0, 24, and 72 h), circulating monocytes were isolated and subjected to the subsequent experiments. Counter plots with quantitative bar charts showed the proportion of MHC-II^−^S100A9^+^ monocytes at different time points after CLP operation (**a**). Representative confocal immunofluorescence images of *S100A9*^+^ monocytes in each group (Scale bar = 50 μm, 20 μm) (**b**). Western blotting analysis indicated the protein expression of S100A9 at various time points (**c**). **d–g** PBMCs and plasma were collected from WT mice subjected to CLP surgery across different sampling time points (0, 24, and 72 h). Histogram and quantitative bar charts indicated the percentage of CD3^+^ cells at different time points after CLP surgery (**d**). Counter plots with quantitative bar charts were compared the proportion of CD3^+^CD4^+^ cells between groups (**e**). Representative counter plots with quantitative bar plots showed CD3^+^CD4^+^Foxp3^+^ Tregs proportion at different time points (**f**). Quantitative bar plots showed circulating levels of IL-2, IL-4, IL-10, IFN-γ, TGF-β and the ratio of IFN-γ to IL-4 in various groups (**g**). Statistics were analyzed by One-way ANOVA with Tukey HSD test for comparison of two groups. Data are shown as means ± SD. ^*^P < 0.05, ^**^P < 0.01, ^***^P < 0.001, ^****^P < 0.0001. CLP cecal ligation and puncture, PBMCs peripheral blood mononuclear cells, WT wild-type, IL interleukins, IFN-γ interferon-γ, TGF-β transforming growth factor-β, DAPI 4′, 6′-diamidino-2-phenylindole, Treg regulatory T cells, MHC major histocompatibility complex, ANOVA analysis of variance, SD standard deviation

To validate above findings at protein level, we collected splenic or circulating monocytes from septic mice, followed by flow cytometry analysis and LSCM examination. Flow cytometry analysis revealed that the percentages of both circulating and splenic MHC-II^−^S100A9^+^ monocytes peaked at 72 h after CLP operation (Fig. 4a, Additional file 1: Fig. S4c). Concomitantly, LSCM examination of monocytes showed marked enrichment of S100A9^+^ monocytes at late phase of sepsis (Fig. 4b). Similar results were also obtained from Western blotting analysis regarding protein level of S100A9 (Fig. 4c). Furthermore, we cultured circulating and splenic monocytes derived from sham or septic mice ex vivo, followed by detection of S100A9 level in the supernatant using ELISA. Consequently, substantial elevation in the release of S100A9 could be consistently observed in both peripheral blood and splenic monocytes from mice at 72 h post-CLP surgery (Additional file 1: Fig. S5). In agreement with the results of our previous study, we observed a transient increase in serum levels of IL-2, IL-4, IL-10, IFN-γ, TGF-β, and IFN-γ to IL-4 ratio at 24 h post-CLP, accompanied by intermediate decline in CD3^+^ T and CD4^+^ T cell proportions in PBMCs. Meanwhile, evident immunosuppression could be confirmed at 72 h post-CLP operation, as supported by dramatically diminished CD3^+^ cells as well as CD4^+^ T lymphocytes, along with substantial expansion of CD3^+^CD4^+^Foxp3^+^ regulatory T cells (Tregs) (Fig. 4d–f). In addition, it was significantly correlated with the reduction of IL-2 and IFN-γ expressions and ratio of IFN-γ to IL-4, accompanied by elevated circulating levels of IL-4, IL-10, and TGF-β (Fig. 4g). Alterations in serum levels of cytokines as well as proportions of T cell subtypes consistently indicated a hyperinflammatory state of CLP mice at early stage of sepsis, with simultaneous upregulation of proinflammatory and anti-inflammatory mediators. Nevertheless, anti-inflammatory cytokines and percentages of immunosuppressive Tregs were substantially augmented upon persistent septic exposure and compensatory increases in proinflammatory cytokines (IL-2 and IFN-γ) at 24 h after septic induction were completely abolished in the meantime, suggesting a shift from proinflammatory phase toward sepsis-induced immunosuppression at later stage of sepsis. These results demonstrate that the proportion of MHC-II^−^S100A9^+^ monocyte appears to be markedly mobilized at late stage of sepsis, which is positively correlated with sepsis-induced immunosuppressive status.

### MHC-II^−^S100A9^+^ monocytes exert immunosuppressive capacity on T cell-mediated immunity

**Fig. 5:**
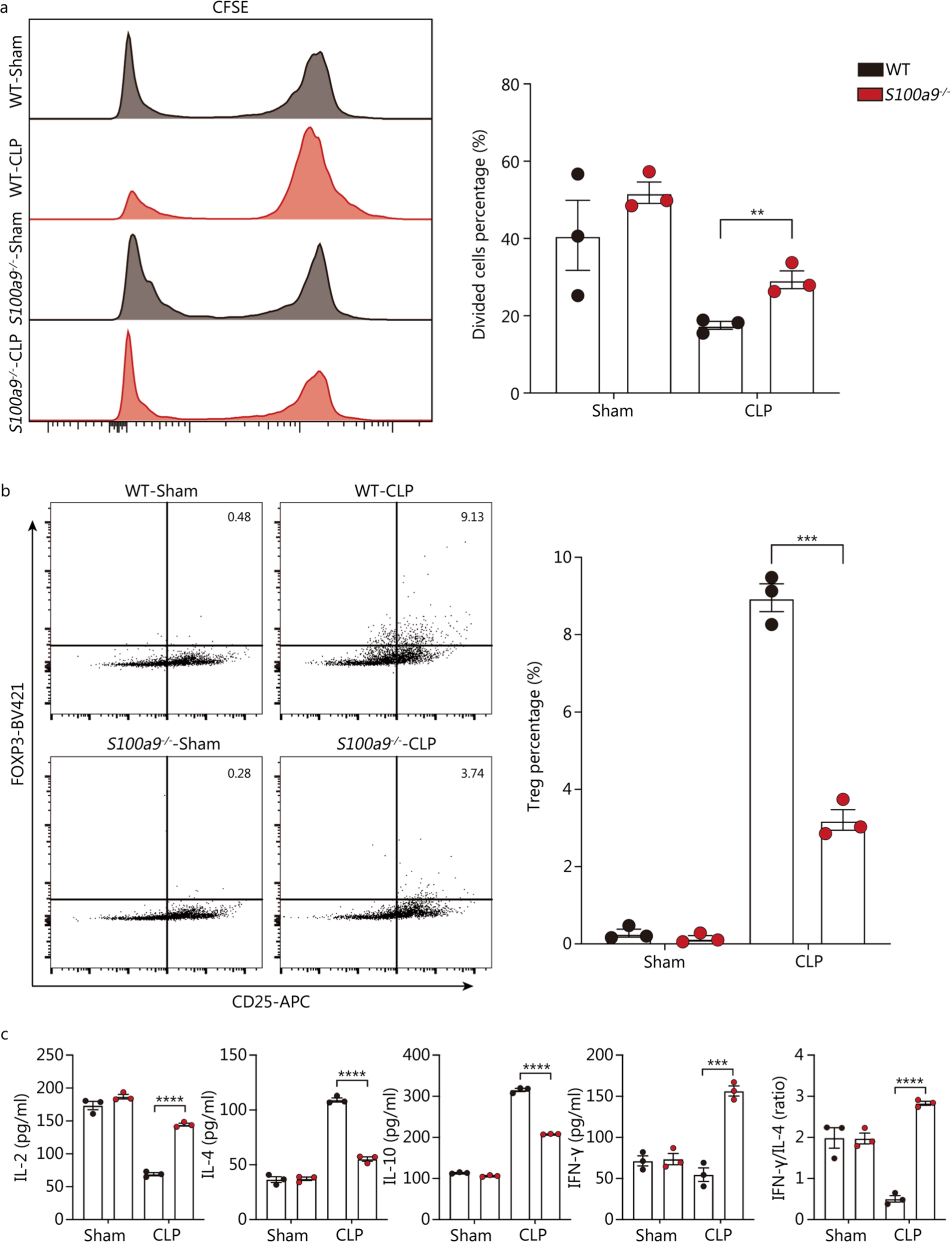
MHC-II^−^S100A9^+^ monocytes exert immunosuppressive function on T cell immunity. Monocytes from spleen of WT or *S100a9*^*−/−*^ mice undergoing CLP surgery for 72 h were isolated, followed by co-culture with CD3/CD28 activated naïve CD4^+^ T cells isolated from unmanipulated murine spleens. Supernatants and CD4^+^ T cells were assayed after 3 d of coculture. **a** Histogram with quantitative bar plot exhibited and compared the proliferative activity of naïve CD4^+^ T cells cocultured with monocytes in each group based on CFSE assay. **b** Contour plots with quantitative bar chart revealed the proportion of CD4^+^CD25^+^Foxp3^+^ Tregs. **c** Quantitative bar charts showed levels of IL-2, IL-4, IL-10, and IFN-γ in the cocultured supernatants, with ratio of IFN-γ to IL-4. Two-way ANOVA with Tukey HSD test was used to determine the statistical significance between groups. Data are shown as means ± SD. ^*^P < 0.05, ^**^P < 0.01, ^***^P < 0.001, ^****^P < 0.0001. MHC major histocompatibility complex, WT wild-type, CLP cecal ligation and puncture, CFSE carboxyl fluorescein succinyl ester staining, Tregs regulatory T cells, IL interleukins, IFN-γ interferon-γ, ANOVA analysis of variance, SD standard deviation

It was noteworthy that S100A9 has been documented to modulate the activity of MDSCs in murine models of sepsis and lymphoma [29, 30]. In consideration of close relationship between MHC-II^−^S100A9^+^ monocytes and immunocompromised state of late sepsis, we investigated if MHC-II^−^S100A9^+^ monocytes exerted immunoregulatory function on T cell-mediated immunity by constructing *S100a9*-deficient (*S100a9*^*−/−*^) mice. By use of carboxyl fluorescein succinyl ester assay, we found that monocytes isolated from CLP mice at 72 h post-operation had dramatically diminished ability in potentiating the proliferation of CD4^+^ T cells in vitro, which could be partially reversed by the *S100a9* deficiency (Fig. 5a). Similarly, monocytes collected from WT septic mice revealed enhanced capacity in driving differential propensity toward Tregs compared with monocytes from sham mice, whereas *S100a9*^*−/−*^ monocytes derived from CLP mice were less effective in potentiating the differentiation of CD4^+^ T cells to Tregs than did WT monocytes of septic mice (Fig. 5b). As expected, splenic monocytes isolated from WT or *S100a9*-deficient CLP mice (72 h) were co-cultured with splenic CD4^+^ T cells harvested from the untreated mice. In comparison to the WT sham group, higher serum levels of IL-4 and IL-10 and decreased secretion of IL-2 and IFN-γ could be detected in the co-cultural medium containing monocytes isolated from WT mice at 72 h post-CLP. Notably, compared to those co-cultured with monocytes from WT septic mice, CD4^+^ T cells co-cultured with *S100a9*^*−/−*^ monocytes upon septic insults were more potent in releasing IL-2 and IFN-γ, with decreased capacity in producing IL-4 and IL-10 (Fig. 5c). The ratio of IFN-γ to IL-4 was higher in supernatants collected from monocytes derived from septic *S100a9*^*−/−*^ mice co-culturing with T lymphocytes, indicating an enhanced phenotypical shift toward helper T cell (Th)1 (Fig. 5c). Thus, these findings suggest that S100A9 is critically involved in the immunosuppressive response of monocytes at late stage of sepsis, implying that MHC-II^−^S100A9^+^ monocytes might manifest analogous features with MDSCs.

### Treatment with Paquinimod ameliorates sepsis-induced immunosuppression by targeting MHC-II^−^S100A9^+^ monocytes

**Fig. 6:**
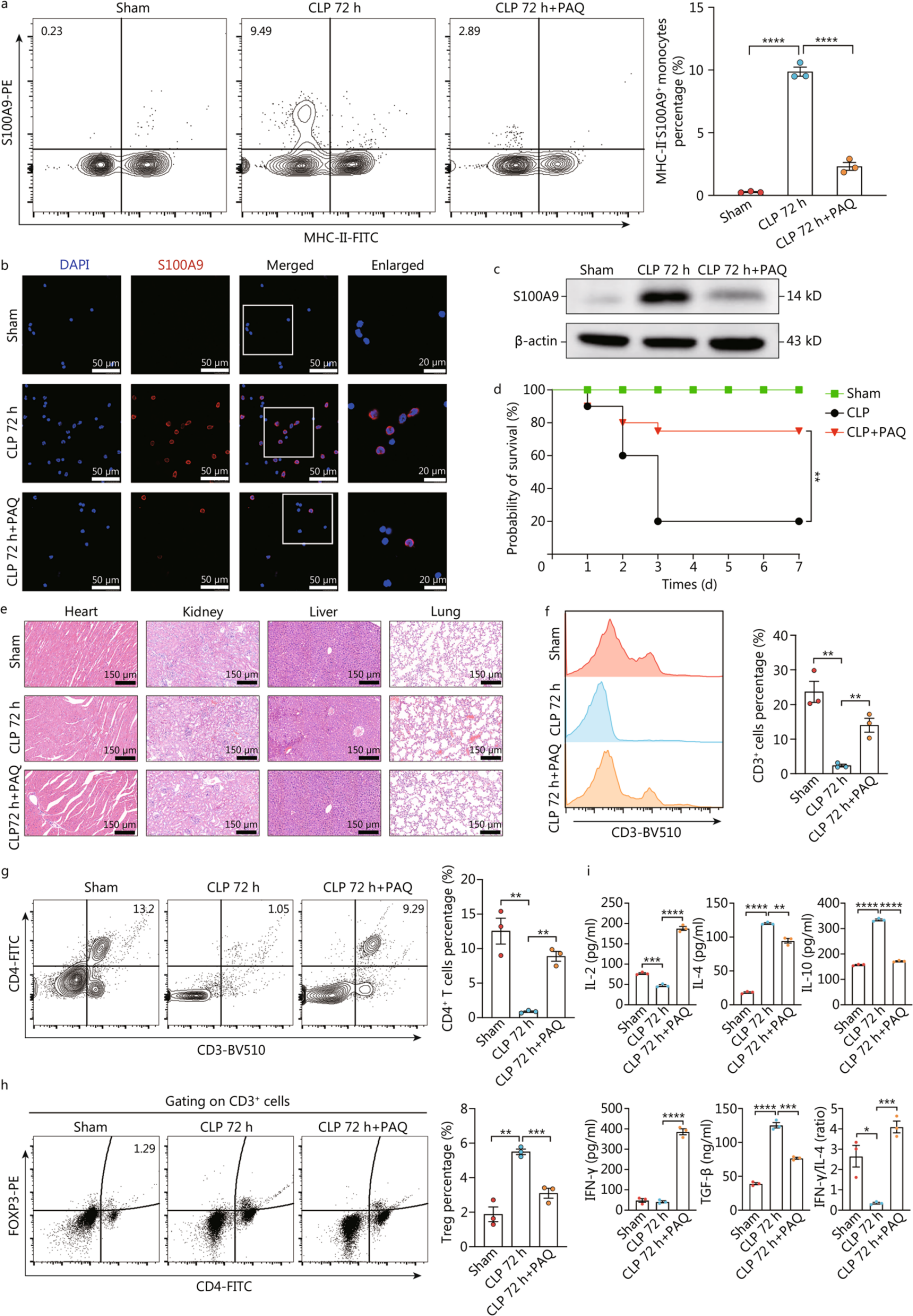
Paquinimod ameliorates sepsis-induced immunosuppression by targeting *S100a*^*high*^ monocytes. **a-c** Mice undergoing CLP surgery were given Paquinimod [(10 mg/(kg·d)] by oral gavage for 3 d or not, followed by isolation of circulating monocytes in sham or CLP mice at 72 h post-operation. Counter plots with quantitative bar charts showed the proportion of MHC-II^−^S100A9^+^ monocytes in various groups (**a**). Representative confocal immunofluorescence images of S100A9^+^ monocytes in each group (Scale bar = 50 μm, 20 μm) (**b**). Western blotting analysis indicated the protein expression of S100A9 (**c**). **d** The survival rates of mice from disparate groups were recorded and compared within 7 d post-CLP surgery, shown by Kaplan–Meier curve. **e** Representative images of HE staining exhibited the pathological alterations in multiple organs of mice, including lung, liver, kidney, and heart (Scale bar = 150 μm). **f-i** PBMCs and plasma were collected from CLP mice treated with or without Paquinimod. Histogram and quantitative bar charts indicated the percentage of CD3^+^ cells in different groups (**f**). Counter plots with quantitative bar charts were compared the proportion of CD3^+^CD4^+^ cells between groups (**g**). Representative counter plots with quantitative bar plots showed CD3^+^CD4^+^Foxp3^+^ Tregs proportion (**h**). Quantitative bar plots showed and compared circulating levels of IL-2, IL-4, IL-10, IFN-γ, TGF-β and the ratio of IFN-γ to IL-4 across each group (**i**). Statistics were by One-way ANOVA with Tukey HSD test for comparison of two groups (**a**, **f–i**). Statistics were calculated using survival curve comparison with log-rank test (**d**). Data are shown as means ± SD. ^*^P < 0.05, ^**^P < 0.01, ^***^P < 0.001, ^****^P < 0.0001, One-way ANOVA, was performed in (**a, f–i**). CLP cecal ligation and puncture, MHC major histocompatibility complex, HE hematoxylin–eosin, PBMCs peripheral blood mononuclear cells, IL interleukins, IFN-γ interferon-γ, ANOVA analysis of variance, SD standard deviation, PAQ Paquinimod

Thereafter, we interrogated the possibility of targeting *HLA-DR*^*low*^*S100A*^*high*^ monocytes for the treatment of sepsis-induced immunosuppression. Paquinimod, also known as ABR-215757 exerts a specific inhibitor that impedes the binding of S100A8/A9 heterodimer to TLR4 [31]. To this end, recent reports have documented the beneficial effect of Paquinimod in ameliorating CLP-induced and acute kidney injury-related proinflammatory responses [32, 33]. Additionally, Paquinimod have been shown to enhance the restoration of aberrant immune response elicited by severe acute respiratory syndrome coronavirus 2 infection and facilitate the elimination of virus, implying its potential application in modulating immune dysregulation [34]. To evaluate the impact of Paquinimod on sepsis-induced immunosuppression, mice underwent CLP procedures were given Paquinimod [(10 mg/(kg·d)] by oral gavage for 3 d [33]. As shown in Fig. 6a–c, treatment with Paquinimod abrogated the upregulation of MHC-II^−^S100A9^+^ monocytes at the late phase of sepsis, as indicated by cytometry, LSCM, and Western blotting, respectively. Similar to the observations of previous studies, we demonstrated that treatment with Paquinimod could attenuate multiple organ injuries, thereby improving the survivals of septic mice (Fig. 6d, e). Simultaneously, Paquinimod treatment was confirmed to enhance immune response of mice with sepsis and partially reversed the immunosuppressive status at late sepsis. Compared with septic mice, elevated numbers of CD3^+^ cells and CD4^+^ T lymphocytes could be noted in septic mice receiving Paquinimod for 3 d, together with decreased proportion of Tregs (Fig. 6f–h). In addition, consecutive administration of Paquinimod was associated with increased circulating levels of IL-2 and IFN-γ as well as ratio of IFN-γ to IL-4, indicating a restored T cell immune response and propensity toward Th1. By treating CLP mice with Paquinimod, circulating levels of IL-10 and TGF-β were obviously decreased (Fig. 6i). Therefore, the above results implicate that pharmacological inhibition of S100A9 using Paquinimod can ameliorate immune dissonance caused by sustainable septic exposure, suggesting the therapeutic potential of targeting *S100A*^*high*^ monocytes for the management of sepsis-induced immunosuppression.

## Discussion

The present study interrogating immune cell landscape of sepsis indicated a substantial heterogeneity of monocyte compartment across disparate septic patients. Specifically, it showed upregulation of distinct monocyte subsets in different cohorts, in which *HLA-DR*^*low*^ and *HLA-DR*^*high*^ monocytes were separately enriched in discriminative clusters of septic patients. By combining single-cell transcriptomics of murine model of sepsis, we identified a unique yet conserved monocyte subcluster characterized by dampened expression of HLA-DR and elevated level of S100A family genes, and it was significantly associated with late-stage of sepsis and immune depression. To interrogate our findings, the results were validated at protein level by in vivo experiments, linking the exploratory investigations on single-cell transcriptomics to functional phenotypes. Moreover, we noticed that *S100A9*^*high*^ monocytes exerted profound immunosuppressive function on CD4^+^ T cell immune response reminiscent of MDSCs, inhibition of which served as a potential therapeutic strategy for treating sepsis-induced immunosuppression. Therefore, this integrative, cross-species scRNA-seq analysis comprehensively deciphers monocyte heterogeneity in the setting of sepsis, providing insight into the cellular and molecular basis of sepsis-induced immunosuppression.

Monocyte reprogramming represents one of the significant hallmarks in sepsis-induced immunosuppression, and monocytes exhibit down-regulated responsiveness to a secondary bacterial pathogen following an initial bacterial challenge, also known as ‘endotoxin tolerance’ [35, 36]. Accordingly, it has been well accepted that circulating monocyte derived from septic patients has a diminished capacity to mount proinflammatory response, as evidenced by decreased production of tumor necrosis factor-α, IL-1α, IL-6, and IL-12 in response to ex vivo lipopolysaccharide stimulation. Nevertheless, its ability to release anti-inflammatory cytokines is unaffected or enhanced, including IL-1 receptor antagonist and IL-10 [37]. Monocytes from septic patients are found to have dampened expression of HLA-DR, a functional marker expressing on various myeloid cells [12, 38]. These features indicate a phenotypical shift of monocyte from proinflammatory subtype to an anergic yet dysfunctional subpopulation upon septic challenge [37, 39]. To this end, the clinical significances of mHLA-DR expression in predicting mortality and nosocomial infection have been validated in multiple cohorts involving patients with severe sepsis or septic shock [12, 40–43]. A RCT conducted by Meisel et al. [44] adopted mHLA-DR level to stratify septic patients with immunosuppression and to guide therapeutic efficacy of immunoadjuvant agent (granulocyte–macrophage colony-stimulating factor). By applying trajectory clustering methodologies, recent studies by Bodinier et al. [45] and Leijte et al. [46] introduced novel mHLA-DR trajectory endotypes among patients with sepsis or septic shock based on mHLA-DR measurement over the first week upon ICU admission, in which they consistently found that septic patients with no improvement or reduction of mHLA-DR expression during the first week showed significantly aggravated clinical prognosis, including elevated mortality rate, increased risk of secondary infection as well as prolonged length of stay in both hospital and ICU. In parallel with these observations, our study identified elevated *CD14*^*+*^*HLA-DR*^*low*^ monocytes in septic patients, which were not observed in healthy controls. Correspondingly, several studies reported that expanded proportion of *HLA-DR*^*low*^ monocytes was positively correlated with an elevated risk of post-traumatic infection and lethal outcome among patients with septic shock [12]. In addition, *CD14*^*+*^*HLA-DR*^*low/−*^ monocytes has been linked to tumor-induced immunosuppression, in association with unfavorable clinical outcomes as well as unresponsiveness to immunotherapeutic agents among cancer patients [47]. As sole use of mHLA-DR expression might render limitations under certain circumstances, Venet et al. [48] proposed a novel yet universal immune monitoring panel incorporating mHLA-DR expression measured by flow cytometry and messenger RNA levels of S100A9, CD3 as well as CD74 for the prediction of acquired immunodeficiency among critically ill patients regardless of etiologies, which revealed encouraging results. Likewise, both the soluble form (sCD163) and the cell membrane-associated form (mCD163) of CD163 have been well characterized as biomarkers in discriminating between septic patients and non-septic patients upon ICU admission [49–51]. A recent study confirmed that the expression level of CD163 on monocyte, determined by median fluorescence intensity, possessed prominent capacity for the prediction of worsening clinical prognosis among patients with sepsis, which represented a well-known hallmark of anti-inflammatory (M2) polarization [52]. In consideration of findings that two dysfunctional monocyte subtypes had been identified in the present study, including *HLA-DR*^*low*^*S100A*^*high*^ and *HLA-DR*^*low*^*CD163*^*high*^ monocytes, it implied a novel yet alternative approach for monitoring sepsis-induced immunosuppression using proportion of these two subtypes. In light of this, multiple methods have been adopted to verify the efficacy of *HLA-DR*^*low*^*S100A*^*high*^ percentage in predicting immunodeficiency, whereas the possibility of applying *HLA-DR*^*low*^*CD163*^*high*^ monocytes as indicators requires further evaluation. Although scRNA-seq technique represents a discovering tool requiring subsequent validation, our study opens novel avenue regarding combined use of well-studied biomarkers mHLA-DR and S100A9 based on flow cytometry analysis, further enhancing accuracy and efficacy of immune monitoring approach used in routine care of septic patients.

Intriguingly, we observed an enrichment of *CD14*^*+*^*HLA-DR*^*high*^ monocytes in two subclusters of septic patients, in which *HLA-DR*^*low*^*S100A*^*high*^ and *HLA-DR*^*low*^*CD163*^*high*^ monocytes were not or intermediately upregulated. This divergency with respect to the distinct enrichment of monocyte subtypes in disparate cohorts was thought to largely depend on disease progression during the course of sepsis, since similar findings have been recently reported in COVID-19 patients [24, 53]. By applying both single-cell proteomics and transcriptomics, their results confirmed that transient activation of *CD14*^*+*^*HLA-DR*^*high*^*CD11c*^*high*^ (*HLA-DRA*^*high*^*CD83*^*high*^) monocytes could be noted only in mild or moderate COVID-19 patients at early timepoint, while *HLA-DR*^*low*^*S100A*^*high*^ monocytes was demonstrated to dominate in severe cases at late phase [24]. We therefore speculated that inflammatory monocytes and defective monocytes might be enriched at early phase and late phase upon onset of sepsis, respectively. It suggests that phenotypical shift from functional to dysfunctional monocytes contributes to the development of sepsis-induced immunosuppression. Concurrently, this hypothesis has been parallelly validated in the experimental model of sepsis, as supported by a causal relationship between proportion of MHC-II^−^S100A9^+^ monocytes and immunosuppressive status of CLP mice. Given that, these data implicate a novel sepsis endotypes on the basis of functional status of monocytes, in which increased proportion of *CD14*^*+*^*HLA-DR*^*high*^ monocytes represents an early stage of sepsis with hyperinflammatory response, whereas patients with elevated *HLA-DR*^*low*^*S100A9*^*high*^ monocytes are evident by immune depression at late stage of sepsis.

In addition to mHLA-DR, another significant feature of dysfunctional monocytes is the enhanced expression of S100A family genes. As an indispensable component of damage-associated molecular patterns, also known as alarmins, S100 proteins are released from various myeloid cells as a result of stress or cell death in response to microbial infection, acting as secondary mediators to amplify innate immunity via binding with pattern-recognition receptors [54]. However, uncontrolled and sustained production of alarmins can initiate a vicious cycle, resulting in detrimental effect on host immune response and even organ failure [34]. S100A8/A9 is the well investigated S100 proteins so far, it constructs non-covalently associated heterodimers expressing in neutrophils, myeloid derived DCs, and monocytes [55]. Since S100A8/A9 represents an endogenous ligand of TLR4, it has been manifested to mediate inflammatory cascades during sepsis [56]. Of note, a pioneering cohort study by Fontaine et al. [57] initially proposed S100A9 messenger RNA level measured at later stage of disease (days 7–10) as an indicator for identifying septic patients at higher risk of developing hospital-acquired infection. Meanwhile, an increased serum level of S100A8/A9 was demonstrated to correlate with a higher mortality rate in patients with septic shock, as excessive production of S100A8/A9 formed a positive feedback loop exacerbating sepsis-induced hyperinflammation [33, 58]. To this end, recently published studies demonstrate the phrenological inhibition of S100A8/A9 using Paquinimod, a specific inhibitor that impedes the binding of S100A9 to TLR4 significantly ameliorates sepsis-induced hyperinflammation, in turn improving survival rate of septic mice [32, 33]. Herein, our study confirmed the protective effect of S100A9 blockade on sepsis, as evidenced by alleviation of multiple organ injuries and decreased mortality rate in CLP mice receiving Paquinimod via oral gavage. Likely, emerging evidence suggests that S100A8/A9 exerts regulatory function in host immune response [54, 59]. It showed that sustained exposure of S100A8/S100A9 hindered the differentiation and antigen presentation capacity of DCs, thereby leading to attenuated T cell response in allergic contact dermatitis [60]. Indeed, our data revealed a direct relationship between proportion of S100A9^+^ monocytes and immunosuppressive status of septic mice, also indicated by the inhibitory experiments, in which administration of Paquinimod abolished the increased of S100A9^+^ monocytes at late stage of sepsis and mitigated sepsis-induced immune dysregulation. These results further extend the rationale of S100A9 blockade using Paquinimod for the treatment of sepsis-induced immunosuppression.

Notably, our results showed that *HLA-DR*^*low*^*S100A*^*high*^ monocytes shared identical features with previously described MS1 monocytes in Reyes’s study, as evidenced by high expression of suggestive transcripts of MS1 monocytes, including *PLAC8*, *CLU*, and *RETN*, all of which were proven to positively correlate with deleterious clinical outcomes among septic patients [20, 61]. As MS1 monocytes possess immunosuppressive capacity, *HLA-DR*^*low*^*S100A*^*high*^ monocytes may closely resemble monocytic MDSCs [28]. In the current study, the functional annotation of the upregulated genes in *HLA-DR*^*low*^*S100A*^*high*^ monocytes as well as cell–cell communication analysis based on predicated ligand-receptor pairs further underpinned this notion. More importantly, S100A9 were confirmed to enhance both the number and suppressive function of MDSCs in several pre-clinical studies, inhibition of which reportedly restored CD8^+^ T cell-mediated anti-tumor responses in lymphoma and prevented repressor activity of MDSCs in sepsis, prompting us to investigate the relationship between *S100A9*^+^ monocytes and MDSCs [29, 62]. Consecutive studies by Gazzar et al. [62, 63] showed that intracellular S100A9 enhanced assembly of signal transducer and activator of transcription 3-CCAAT/enhancer-binding protein alpha in an IL-10 dependent manner, which in turn upregulated expression of immune repressor mediators including microRNA-21 and microRNA-181b, thereby facilitating expansion and immunosuppression of MDSCs during late sepsis. They also demonstrated that both IL-10 mediated signaling and downregulation of long non-coding RNA Hotairm1 drove the S100A9 translocation from cytosol to the nucleus, representing the prerequisite of immunoregulatory effect of intracellular S100A9 [64, 65]. By coculturing splenic monocytes with naïve CD4^+^ T cells, we found that monocytes derived from septic mice at late stage after CLP displayed potent inhibitory effect on T cell proliferation and augmented differentiation toward Th2 as well as Tregs in comparison to that of monocytes derived from the sham group, and all of which could be partially rescued by S100A9 depletion. These results collectively demonstrate the immunoregulatory property of S100A9 in monocytes, thereby providing phenotypic characterization of human monocytic-MDSCs in sepsis. Our study also highlights the role of intracellular S100A9 protein that acts as a transcription chaperone or an epigenetic mediator in augmenting immunosuppressive function of phagocytes and MDSCs, rather than an extracellular proinflammatory mediator. Since holistic deletion of *S100a9* in mice can inevitably render bias and restrict us from conducting in vivo experiments, conditional knockout of *S100a9* in monocytes seems to be the ideal subject in further study to rule out the influencing factors associated with other cell types, thereby providing more convincing results.

Several limitations should be considered when interpreting our data. Given the lack of detailed information regarding baseline characteristics of individual patients, we were unable to establish the causal link between *HLA-DR*^*low*^*S100A*^*high*^ monocytes and sepsis-induced immunosuppression. Also, a prospective cohort of septic patients is missing to solidly validate the presence of *S100A*^*high*^ monocytes and its direct association with the disease progression during sepsis. Thus, future scRNA-seq studies may incorporate septic patients exquisitely divided by disease severity and by the time after onset of sepsis. Meanwhile, merely transcriptomic data of human sepsis was analyzed in the current study. From this, multi-omics complementary technologies including single-cell proteomics may do great help to extend our findings and to verify these results. Additionally, we did not carry out in-depth interrogation into the exact ontogeny of the *HLA-DR*^*low*^*S100A*^*high*^ monocytes. To achieve this, further analysis should be adopted to comprehensively delineate the key transcriptional factors driving the differentiation of this subset, which might represent the prerequisite for uncovering its relationship with MDSCs in sepsis. Finally, although we interrogated the potential role of Paquinimod in ameliorating sepsis-induced immunosuppression, its effect on TLR4 in the bone marrow requires to be explored. Therefore, further experiments incorporating other TLR4 blockers are needed to determine whether the effects observed are specifically related to Paquinimod’s interactions with TLR4, uncovering the mechanisms by which Paquinimod might be restoring homeostasis.

## Conclusions

Our results identify a conserved monocyte subset characterized by high expression of S100A family genes and low expression of HLA-DR, namely *HLA-DR*^*low*^*S100A*^*high*^ monocytes, and it is predominantly enriched together with immunosuppressive response in late sepsis. Therefore, this study links a striking occurrence of dysfunctional monocyte subtype to sepsis-induced immunosuppression, which might not only deepen our understanding with regard to the pathogenesis of sepsis, but improve the identification of novel immune monitoring indicators and development of personalized immunomodulatory therapies for sepsis.

## Supplementary Information

**Additional file 1: Fig. S1** Characteristics of the dataset and markers of cell subsets. **Fig. S2** Trajectory and cell–cell interaction analyses of monocyte subtypes. **Fig. S3** ScRNA-seq analysis reveals monocyte heterogeneity in septic patients with ARDS. **Fig. S4** identification of splenic *S100a*^*high*^ monocytes in murine sepsis. **Fig. S5** S100A9 release of circulating and splenic monocytes upon septic challenge. **Table S1** Clinical characteristics of enrolled patients. **Table S2** Composition of clinical entities in each cluster.

## Data Availability

The raw data that support the findings of this study are available from the corresponding author (Yong-Ming Yao) upon reasonable request and through collaborative investigations. All other data needed to evaluate the conclusions in the paper are presented in the paper or the Supplementary Materials.

## Abbreviations

ANOVA: Analysis of variance
CLP: Cecal ligation and puncture
COVID-19: Coronavirus disease 2019
DCs: Dendritic cells
DEGs: Differentially expressed genes
ELISA: Enzyme-linked immunosorbent assay
ICU: Intensive care unit
IFN-γ: Interferon-γ
IL: Interleukin
LSCM: Laser scanning confocal microscopy
MDSCs: Myeloid-derived suppressor cells
MHC: Major histocompatibility complex
mHLA-DR: Monocyte human leukocyte antigen DR
NK: Natural killer
PBMCs: Peripheral blood mononuclear cells
PBS: Phosphate buffer solution
RCTs: Randomized controlled trials
scRNA-seq: Single-cell RNA sequencing
SD: Standard deviation
TGF-β: Transforming growth factor-β
TLR: Toll-like receptor
Tregs: Regulatory T cells
UMAP: Uniform manifold approximation and projection
UTI: Urinary tract infection
WT: Wild-type
