# Supplementary material for: Single-cell transcriptome profiling of sepsis identifies HLA-DRlowS100Ahigh monocytes with immunosuppressive function
Source: Mil Med Res. 2023 Jun 19;10:27. doi: 10.1186/s40779-023-00462-y (PMC10278311; doi:10.1186/s40779-023-00462-y)
Supplement: Supplementary file 1 — Additional file 1: Fig. S1 Characteristics of the dataset and markers of cell subsets. Fig. S2 Trajectory and cell–cell interaction analyses of monocyte subtypes. Fig. S3 ScRNA-seq analysis reveals monocyte heterogeneity in septic patients with ARDS. Fig. S4 identification of splenic S100ahigh monocytes in murine sepsis. Fig. S5 S100A9 release of circulating and splenic monocytes upon septic challenge. Table S1 Clinical characteristics of enrolled patients. Table S2 Composition of clinical entities in each cluster. [file 40779_2023_462_MOESM1_ESM.pdf]

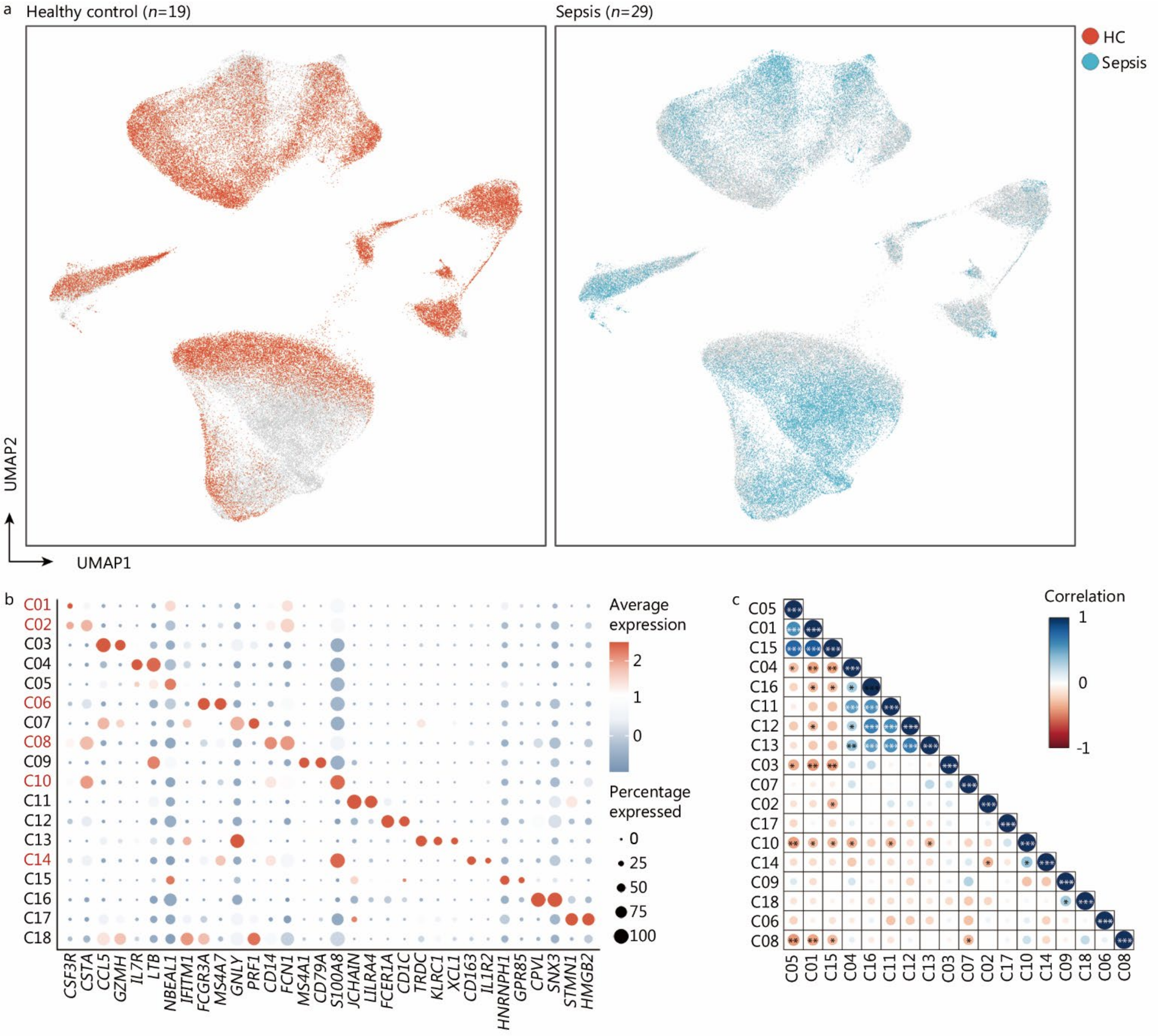

Fig. S1 Characteristics of the dataset and markers of cell subsets. **a** UMAPs visualization of scRNA-seq profiles according to clinical entities. **b** Dot plots of the intersection of the top 2 marker genes across disparate immune cell subsets. **c** Correlation heatmap showed the relationship in terms of proportional alteration among each cluster. Statistics were analyzed by Pearson's correlation test. $^{*}P < 0.05$, $^{**}P < 0.01$, $^{***}P < 0.001$, $^{****}P < 0.0001$. UMAPs uniform manifold approximation and projections, scRNA-seq single-cell RNA sequencing, HC healthy control

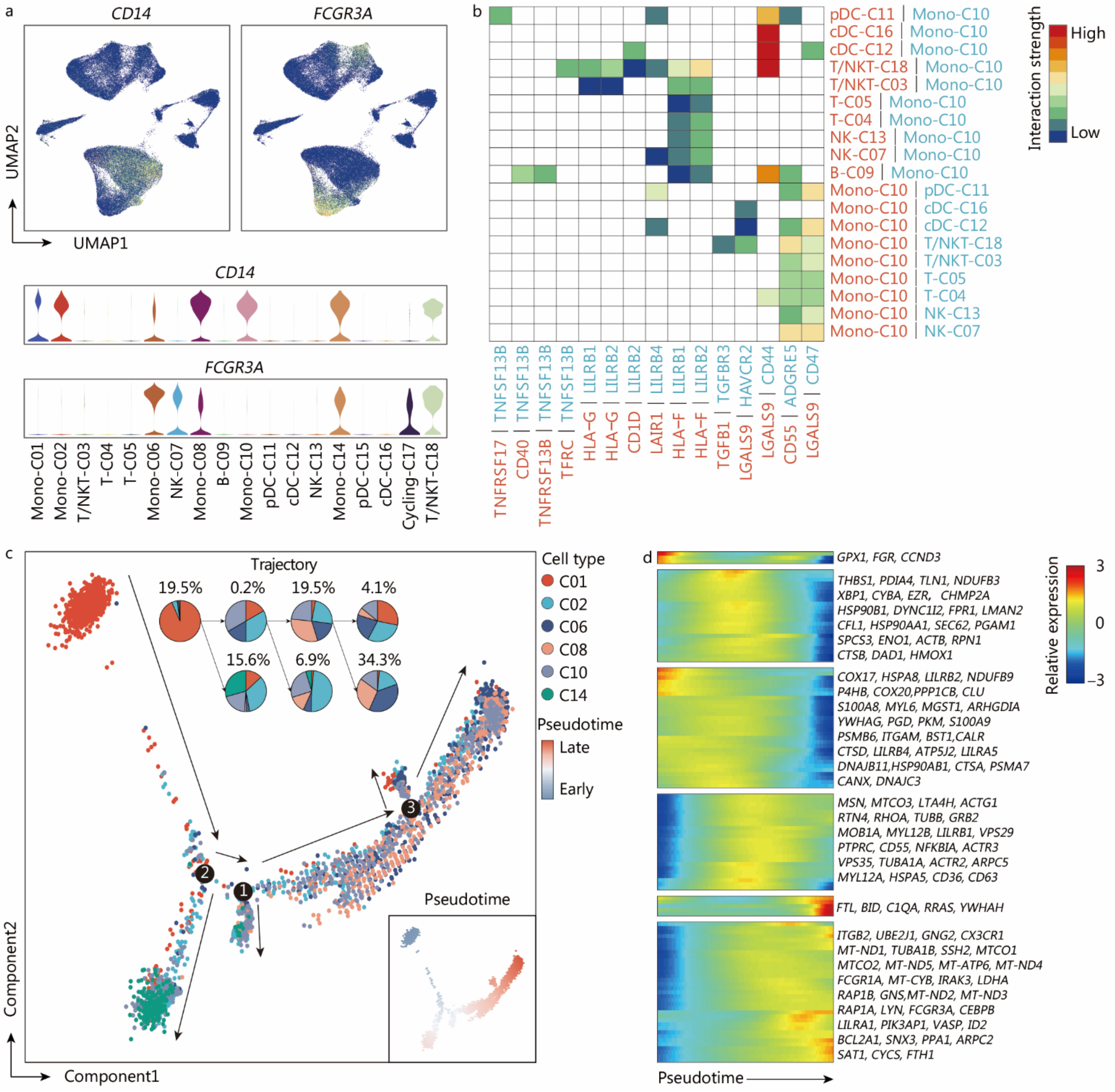

Fig. S2 Trajectory and cell-cell interaction analyses of monocyte subtypes. **a** UMAPs (upper panel) and violin plots (lower panel) showed expression level of key lineage markers of classical monocyte (*CD14*) and non-classical monocyte (*FCGR3A*/*CD16*) in each immune cell subpopulation. **b** Histogram revealed the cell-cell communication between C10 and other immune cell types, based on selected ligand-receptor pairs inferred by 'CellPhoneDB', with color of grey indicating no statistical significance. **c** The developmental trajectory of colored-coded monocyte subtypes by the clusters and pseudo-time. Putative trajectory for cell transition states of monocyte, with proportion of each subcluster. **d** Heatmap displayed the dynamic transitions in expression level of DEGs across monocytes

subclusters along with the pseudo-time. UMAPs uniform manifold approximation and projections, DEGs differentially expressed genes, Mono monocyte, DC dendritic cells, NK natural killer, TNF tumor necrosis factor

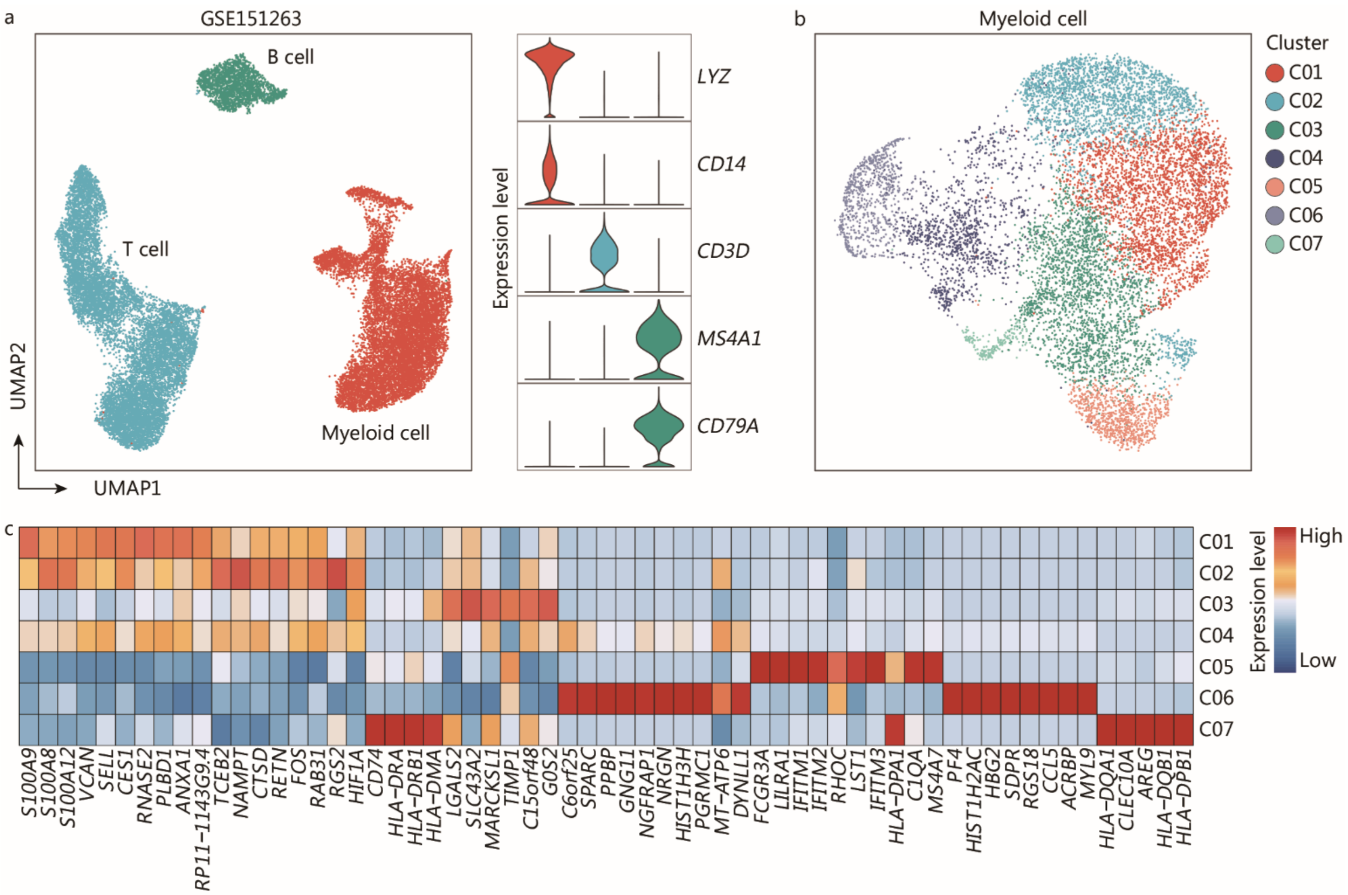

Fig. S3 ScRNA-seq analysis reveals monocyte heterogeneity in septic patients with ARDS. **a** UMAP visualization (left panel) and violin plots (right panel) displayed the annotation and color-coded clusters of main immune cell types in septic patients with or without ARDS. **b** UMAP plot exhibited clustering analysis of monocyte subtypes. **c** Heatmap showed relative expression levels of top DEGs across subclustered monocyte subsets. scRNA-seq single-cell RNA sequencing, ARDS acute respiratory distress syndrome, UMAPs uniform manifold approximation and projections, DEGs differentially expressed genes

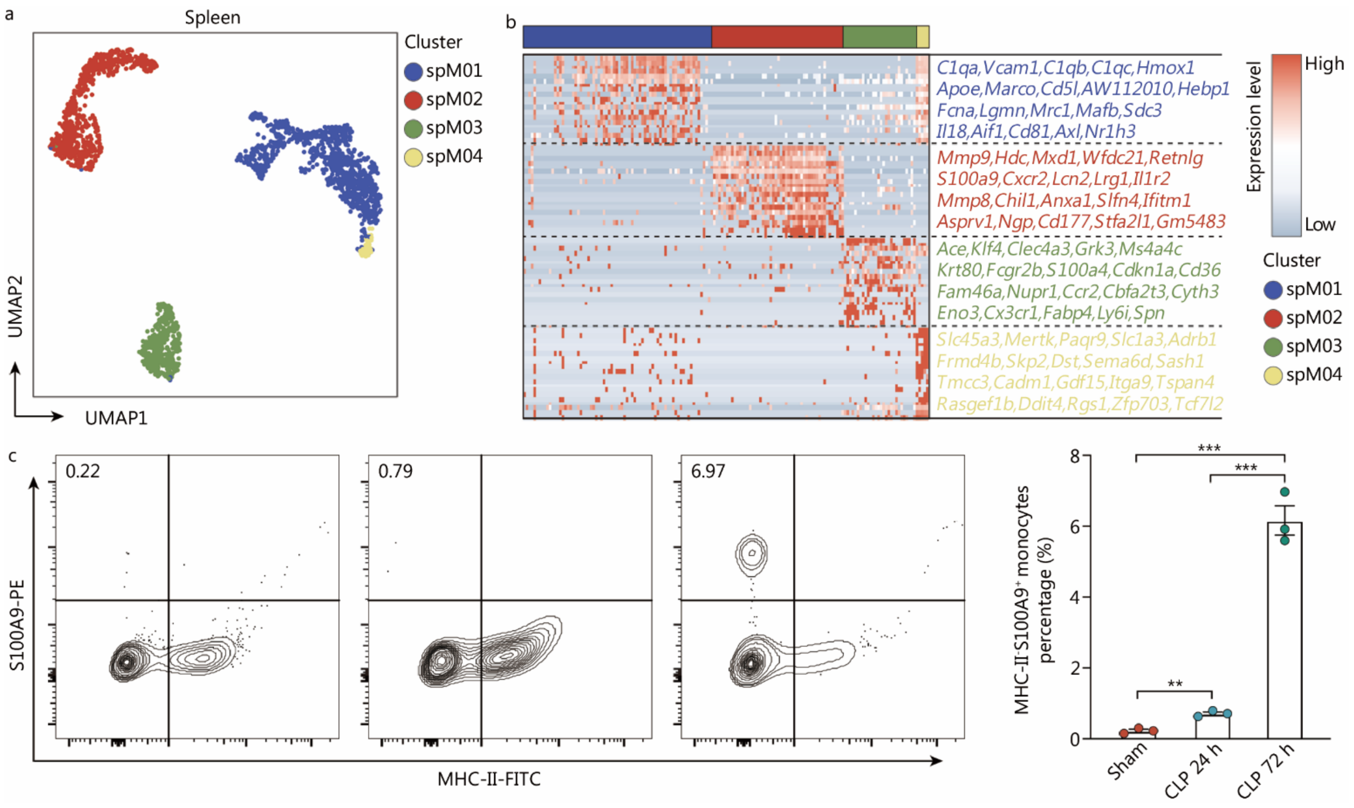

Fig. S4 Identification of splenic *S100a*^{high} monocytes in murine sepsis. **a** UMAP showed subclusters of splenic monocytes in murine sepsis. **b** Heatmap showed the relative expression of DEGs among all monocyte subpopulations. **c** Representative counter plots with quantitative bar charts indicated the proportion of MHC-II^{-} S100A9^{+} monocytes in spleen at various time points after CLP operation. Statistics were analyzed by One-way ANOVA with Tukey HSD test for comparison of two groups. Data are shown as means ± SD. $^{*}P < 0.05$, $^{**}P < 0.01$, $^{***}P < 0.001$, $^{****}P < 0.0001$. MHC major histocompatibility complex, UMAP uniform manifold approximation and projection, DEGs differentially expressed genes, CLP cecal ligation and puncture, ANOVA analysis of variance, SD standard deviation

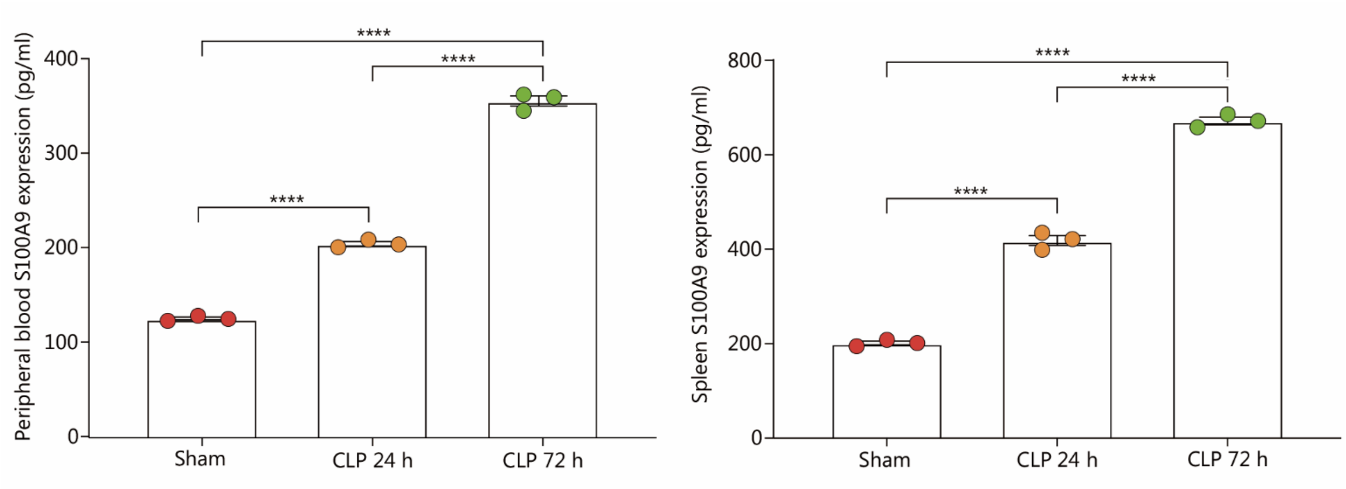

Fig. S5 S100A9 release of circulating and splenic monocytes upon septic challenge. One-way ANOVA with Tukey HSD test was used to determine the statistical significance between groups. Data are shown as means ± SD. $^{*}P < 0.05$, $^{**}P < 0.01$, $^{***}P < 0.001$, $^{****}P < 0.0001$. CLP cecal ligation and puncture, ANOVA analysis of variance, SD standard deviation

Table S1 Clinical characteristics of enrolled patients

Group	Clinical entities	Age, Median year (IQR)	Male, *n* (%)	SOFA, Median (IQR)	Patient ID
Control	Healthy control (*n* = 19)	57 (51 – 66)	7 (37)	---	P18F<br>P02H<br>P09H<br>C2P13F<br>C2P16H<br>C2P01H<br>C2P10H<br>C2P19H<br>C2P05F<br>P06F<br>P07H<br>P04H<br>P13H<br>P17H<br>C2P15H<br>P15F<br>P20H<br>P08H<br>C2P07H
Sepsis	Int-URO (*n* = 7)	71 (54 – 75)	1 (14)	2 (2 – 3)	C2P19<br>C2P15<br>C2P18<br>C2P12<br>C2P11<br>P05<br>P09
	URO (*n* = 10)	69 (55 – 75)	6 (60)	4 (2 – 6)	C2P13<br>C2P10<br>C2P05<br>C2P09<br>C2P02<br>C2P16<br>C2P01<br>P04<br>P08<br>C2P21

Bac-SEP (*n* = 4)	64 (58 – 73)	4 (100)	2 (0 – 3)	E1<br>E25<br>E12<br>E16
ICU-SEP (*n* = 8)	63 (59 – 68)	6 (75)	4 (3 – 4)	P640<br>P662<br>P669<br>P670<br>P672<br>P671<br>P633<br>P636

IQR interquartile range, *SOFA* Sequential organ failure assessment, *Int-URO* urinary tract infection (UTI) with intermediate organ dysfunction, *URO* UTI with evident organ dysfunction, *Bac-SEP* bacterial sepsis in hospital wards, *ICU-SEP* admission to the intensive care unit (ICU) with sepsis

Table S2 Composition of clinical entities in each cluster

Cluster	Patient ID	Clinical entities
G1	P04	URO
	P08	URO
	P13H	Healthy control
	P15F	Healthy control
	P04H	Healthy control
	P07H	Healthy control
	P06F	Healthy control
	P08H	Healthy control
G2	C2P16	URO
	E12	Bac-SEP
	C2P10	URO
	P640	ICU-SEP
	P636	ICU-SEP
	E16	Bac-SEP
	P671	ICU-SEP
	E25	Bac-SEP
	P09	Int-URO
	E1	Bac-SEP
	P669	ICU-SEP
G3	C2P05F	Healthy control
	C2P13F	Healthy control
	C2P01H	Healthy control
	C2P10H	Healthy control
	C2P15H	Healthy control
	C2P16H	Healthy control
G4	C2P05	URO
	C2P09	URO
	P05	Int-URO
	C2P11	Int-URO
	P633	ICU-SEP
	C2P13	Int-URO

	C2P18	URO
G5	P17H	Healthy control
	P20H	Healthy control
	C2P01	URO
	P18F	Healthy control
	C2P19	Int-URO
	C2P19H	Healthy control
	P02H	Healthy control
	C2P07H	Healthy control
	P09H	Healthy control
G6	C2P02	URO
	C2P21	URO
	C2P12	Int-URO
	C2P15	Int-URO
	P662	ICU-SEP
	P670	ICU-SEP
	P672	ICU-SEP

Int-URO urinary tract infection (UTI) with intermediate organ dysfunction, *URO* UTI with evident organ dysfunction, *Bac-SEP* bacterial sepsis in hospital wards, *ICU-SEP* admission to the intensive care unit (ICU) with sepsis
